# Efficacy of a Multi-Strain Probiotic Formulation in Pediatric Populations: A Comprehensive Review of Clinical Studies

**DOI:** 10.3390/nu13061908

**Published:** 2021-06-01

**Authors:** Annie Tremblay, Xiaoyu Xu, James Colee, Thomas A. Tompkins

**Affiliations:** 1 Rosell Institute for Microbiome and Probiotics, Lallemand Health Solutions Inc., 6100 Royalmount Avenue, Montreal, QC H4P2R2, Canada; atremblay@lallemand.com (A.T.); jadecher@hotmail.com (X.X.); 2 IFAS Statistical Consulting Unit, University of Florida, P.O. Box 110500, Gainesville, FL 32611-0500, USA; jcolee@gmail.com

**Keywords:** probiotics, Biostime^®^, Probiokid^®^, infants, newborns, children, infantile diarrhea, immune defenses

## Abstract

A probiotic formulation combining *Lactobacillus helveticus* Rosell^®^-52, *Bifidobacterium infantis* Rosell^®^-33, and *Bifidobacterium bifidum* Rosell^®^-71 with fructooligosaccharides, first commercialized in China, has been sold in over 28 countries since 2002. Clinical studies with this blend of strains were conducted mainly in pediatric populations, and most were published in non-English journals. This comprehensive review summarizes the clinical studies in infants and children to evaluate the efficacy of this probiotic for pediatric indications. Literature searches for pediatric studies on Biostime^®^ or Probiokid^®^ (non-commercial name) in 6 international and Chinese databases identified 28 studies, which were classified by indications. Twelve studies show that the probiotic significantly increases the efficacy of standard diarrhea treatment regardless of etiology, reducing the risk of unresolved diarrhea (RR 0.31 [0.23; 0.42]; *p* < 0.0001) by 69%. In eight studies, the probiotic enhanced immune defenses, assessed by levels of various immune competence and mucosal immunity markers (six studies), and reduced the incidence of common infections (two studies). The probiotic improved iron deficiency anemia treatment efficacy (three studies), reducing the risk of unresolved anemia by 49% (RR 0.51 [0.28; 0.92]; *p* = 0.0263) and significantly reducing treatment side effects by 47% (RR 0.53 [0.37; 0.77]; *p* = 0.0009). Other studies support further investigation into this probiotic for oral candidiasis, eczema, feeding intolerance in premature babies, or hyperbilirubinemia in newborns.

## 1. Introduction

*Lactobacilli* and *Bifidobacteria* are commensal bacteria of the human and animal gastrointestinal (GI) tract, and several strains of these genera are widely used as probiotics and in food supplements, or as starter or adjunct cultures in the production of dairy products or other fermented foods. Although *Lactobacilli* have been shown to represent a small proportion of the fully developed and highly diverse adult microbiota, their importance is demonstrated by the association between their modulation and various diseases [1]. *Bifidobacteria* were shown to be among the first colonizers of the healthy infant’s GI tract, and to predominate in the intestinal tract until the transition to a solid food diet, at which point microbiota diversity begins to increase towards the varied composition typically seen in adults [2]. With the increasing awareness of the role of the microbiota and dysbiosis in pediatric health and diseases [3], the use of probiotics is considered among the potential interventions available to target the microbiota in pediatric populations. However, assessing the overall safety and efficacy of probiotics in general for a given indication increases heterogeneity when the analyses combine studies having assessed a variety of probiotic strains or formulations, dosing regimen, and outcome assessment measures [4].

The probiotic formulation reviewed herein is composed of *Lactobacillus* (*L.*) *helveticus* Rosell^®^-52, *Bifidobacterium* (*B.*) *longum* subsp. *infantis* (*B. infantis*) Rosell^®^-33 and *B. bifidum* Rosell^®^-71, with fructooligosaccharides. All three strains were deposited at the Pasteur Institute in the “Collection Nationale de Cultures de Microorganismes” (CNCM), under the numbers CNCM I-1722, CNCM I-3424 and CNCM I-3426, respectively [5,6]. The whole-genome sequences are deposited in the PATRIC database (https://www.patricbrc.org/ (accessed on 31 May 2021)) under the genome identification numbers (Genome ID): 880633.7, 1678.111, and 1678.107, respectively. First marketed in China in 2002, this formulation has since been commercially available in over 28 countries. The innocuity of the included strains is recognized by several authoritative bodies worldwide, where they are included in lists of safe strains for consumption in foods. Probiokid^®^ and the individual strains have been recognized for their safety by the US FDA in the form of a no question letter for notified GRAS status for use in non-exempt infant formula, in Canada by the NNHPD for use in Natural Health Products in infants 3 months old and over, and in China for safe use in food for infants and young children. In addition, the safety of this product is also monitored through a pharmacovigilance program covering both foreign and domestic adverse event cases [7].

The clinical studies conducted with this probiotic formulation have demonstrated its beneficial effects on GI and immune functions in children. With many of the studies published in non-English journals, there is a barrier for results dissemination to the global scientific community. Hence, we undertook this comprehensive literature review summarizing the clinical studies on this probiotic formulation published in North America, Europe, and Asia to provide an overview of the global evidence base available on this probiotic in pediatric populations. In addition, when possible, outcome-specific meta-analyses were conducted on studies harboring similar design and outcome assessment methods, which strengthens the conclusions obtained from individual trials while providing more specificity than the meta-analyses including different probiotic strains or formulations.

## 2. Materials and Methods

The results of this comprehensive review are reported according to the PRISMA guidelines. The protocol of this review was not prospectively registered. Articles of interest were identified by searching for the trade names, Probiokid^®^ (non-commercial name) or Biostime^®^ (合生元^®^), and for the individual strain names and numbers (Supplemental Table S1) using search engines for scientific or medical journal databases up to May 2021. Two independent assessors conducted the searches, screening against inclusion/exclusion criteria, and data extraction (A.T., X.X.). Discrepancies were solved via discussion between the two authors, and a third assessor was consulted as needed for resolution (T.A.T.). Articles in Chinese, although most had an abstract in English, were translated using Google translate and a native speaker (X.X.) performed inclusion/exclusion criteria assessment and data extraction using the original language articles. For the selection process, retrieved articles were screened by title and abstract to identify the clinical trials in pediatric populations and then examined for study design, regardless of the indication. Only studies where controls allowed for a direct assessment of the effect of the probiotic were included. These encompassed studies where the probiotic formulation was used as adjuvant with the active comparator alone used as control, studies comparing with a placebo, another probiotic (e.g., GoldenBifido, Lacidophilin), or a standard of care (e.g., Smecta^®^, iron dextran, phototherapy) as control, or case–control-type studies allowing for a comparison of the effect before and after the probiotic intervention. Study authors were not contacted to provide additional data.

**Figure 1:**
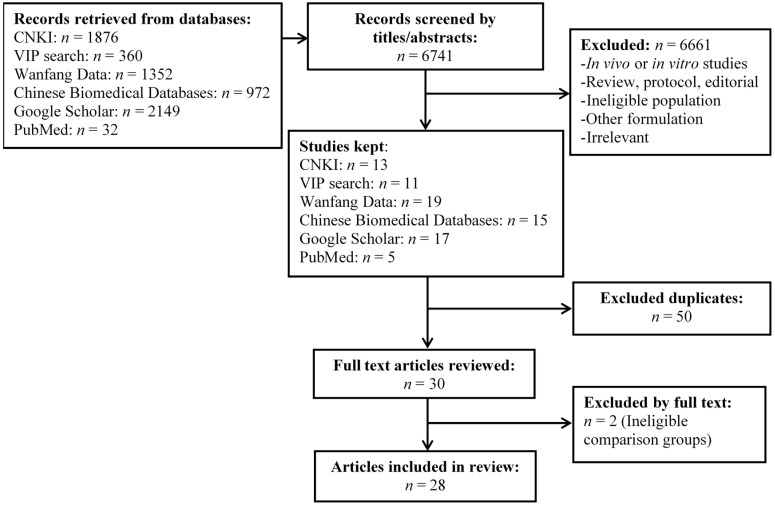
PRISMA flow diagram.

A total of 28 studies in pediatric populations were included and are detailed in this comprehensive review (Figure 1).

The characteristics of the 28 studies were extracted using a tabular format including study dates, population, study design, arms and n, treatment regimen, results, and adverse events (including serious adverse events). All studies were conducted with the same formulation which contains the strains *L. helveticus* Rosell^®^-52, *B. infantis* Rosell^®^-33 and *B. bifidum* Rosell^®^-71, with 750 mg of fructooligosaccharrides per sachet. This formulation is manufactured to ensure a minimal active dose of 3 billion colony-forming units (CFU) per sachet at the end of shelf-life. Considering that reporting standards for the dosage vary between countries, the Chinese or some European studies tend to report the dose at manufacturing, which is higher to maintain the guaranteed minimal active dose per sachet until the end of shelf-life. Therefore, for comparability, dosage information is provided herein based on the number of sachets administered.

After examination of the study characteristics tables, a meta-analysis was conducted if 3 studies or more allowed to calculate the relative risk for a given outcome when similar design, assessment method and data reporting format were used. Meta-analyses were conducted in R version 4.0.2, using the meta package [8,9]. Weighing was calculated using the inverse variance method, the DerSimonian–Laird estimator was used for τ^2^, and the Jackson method was used for confidence interval of τ^2^ and τ. Studies included in meta-analyses were assessed by 2 authors (A.T., X.X.) for the risk of bias (low, unclear, high) [10], and the overall level of bias was assigned for the treatment efficacy outcome used in the meta-analysis. Discrepancies were resolved through discussion, and a third assessor (T.T.) was consulted as needed. For the studies included in the diarrhea outcome meta-analysis, publication bias was estimated using a funnel plot and asymmetry quantification using the Egger’s regression test. The Trim and Fill method by Duval and Tweedie was used to estimate the number of missing studies and these estimated negative studies were added to the meta-analysis as a sensitivity assessment [11,12].

## 3. Results

### 3.1. Classification of Identified Clinical Studies

The reviewed trials were categorized by indication, delineating three broad categories (Table 1). Overall, the identified trials investigated the safety and efficacy of the probiotic at restoring normal GI function in cases of diarrhea of various etiologies (12 studies; summarized in Table 2) and at supporting immune system function and natural defenses (8 studies; summarized in Table 3), mainly by enhancing immune competence (6 studies) and reducing the occurrence or recurrence of common infections (2 studies). In addition, other studies (summarized in Table 4) investigated the role of this probiotic combination in infant eczema (one study), oral candidiasis (one study), nutritional iron deficiency anemia (three studies), necrotizing enterocolitis (one study) and hyperbilirubinemia (jaundice; two studies).

### 3.2. Studies on Diarrhea of Various Etiologies

Twelve studies of similar design were conducted in China on children with diarrhea of various etiologies. A total of 1356 infants were enrolled, of whom 714 received a similar probiotic regimen, provided as an adjuvant to a standard of care. These 12 randomized trials (Table 2) assessed efficacy using the same categorical scale, in accordance with the Chinese national standards for the diagnosis and treatment of diarrhea [40], as follows: a “markedly effective” result is characterized by the frequency and characteristics of the stool returning to normal (<3 bowel movements/day) and symptoms disappearing within 72 h of intervention; an “Effective” result is characterized by a significant improvement in the frequency (<4 bowel movements/day) and characteristics of stool and symptoms within 72 h of intervention; and an “ineffective” result is characterized by the frequency, characteristics of stools and overall symptoms failing to improve within 72 h of intervention.

**Figure 2:**
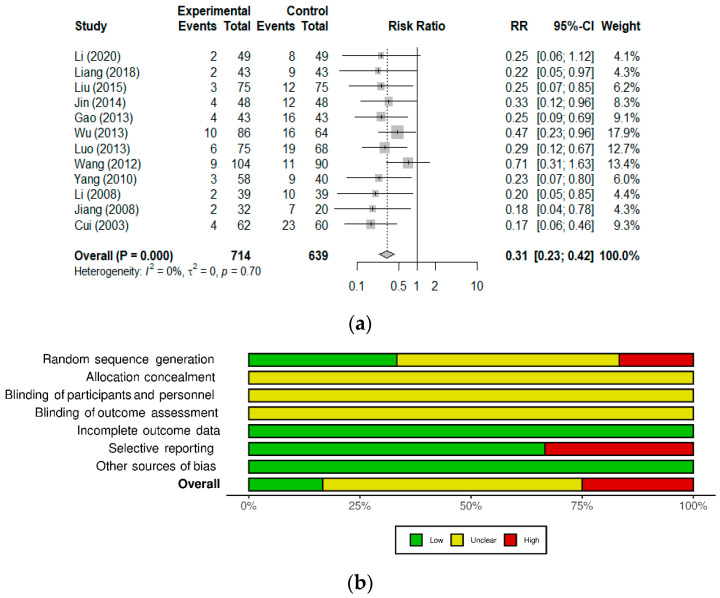
The probiotic formulation used as adjuvant reduced the relative risk of unresolved diarrhea. (**a**) Forest plot of studies with an efficacy outcome (effective rate) for diarrhea of various etiologies. Events refers to the number of cases categorized as inefficient (unresolved diarrhea); Total refers to the number of participants in the Experimental (Probiotic) and Control groups. The 12 studies included in the meta-analysis are detailed in Table 2. (References: Cui, 2003 [22], Li, 2008 [24], Jiang, 2008 [26], Yang, 2010 [25], Wang, 2012 [20], Luo, 2013 [19], Gao, 2013 [15], Wu, 2013 [21], Jin, 2014 [23], Liu, 2015 [18], Liang, 2018 [17], Li, 2020 [16]). (**b**) Mosaic plot showing the risk of bias summary of the studies included in the meta-analysis.

Considering the high level of similarity between the 12 diarrhea studies, we conducted a meta-analysis of the diarrhea treatment outcome (Figure 2a). Overall, probiotic supplementation significantly improved the efficacy of standard diarrhea treatment; pooled results from the 12 studies show a 69% reduction in the RR of unresolved diarrhea (RR 0.31 [0.23; 0.42]; *p* < 0.0001), regardless of etiology. Heterogeneity among studies was assessed using *I*^2^ and τ^2^ and found absent, which was in accordance with the overall RR and 95% CI values being the same using either the fixed- or random-effects models.

**Figure 3:**
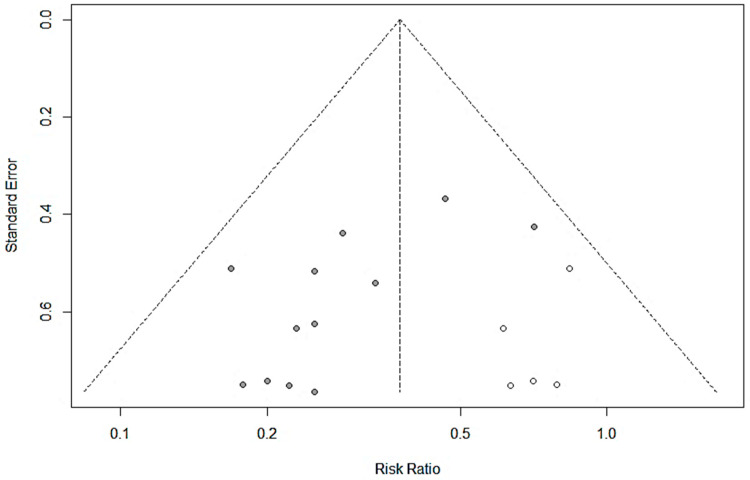
Funnel plot showing the relationship between the relative risk and its standard error for the 12 studies included (filled circles) and the potential 5 negative studies added to correct asymmetry using the Trim and Fill method (empty circles).

For the risk of bias analysis (Figure 2b), most studies included in the meta-analysis were categorized as low or unclear risk [15,16,17,18,20,22,23,24,26], with three studies classified as high risk of bias due to potential randomization or selective reporting issues [19,21,25]. All studies were classified as unclear risk for the allocation concealment and blinding-related domains; a similar trend was observed in a previous systematic review, which could reflect regional publication practices [41]. Publication bias was assessed using the funnel plot method (Figure 3) and asymmetry was found significant (Egger’s regression test; t = −2.84, df = 10, *p* = 0.0176), suggesting the potential occurrence of publication bias. The number of missing studies to correct the asymmetry was estimated to five using the Trim and Fill method by Duval and Tweedie [11,12]. Upon inclusion of 5 negative studies in the meta-analysis (empty circles; total studies = 17), the result remained significant with the reduction in the risk of unresolved diarrhea changing from 69% to 62.5% (RR 0.3753 [0.2867; 0.4913]; *p* < 0.0001).

### 3.3. Studies on Immune Competence and Inflammatory Markers

All studies classified in the immunity and natural defenses indications are detailed in Table 3. At the mucosal interface, secretory immunoglobulin A (sIgA) mediate humoral immunity by regulating the balance between immune exclusion of pathogenic microorganisms and tolerance to commensal bacteria, thereby favoring the establishment of a symbiotic relationship between the gut microbiota and the host [42,43]. At birth, immunoglobulins (Ig), and mainly secretory IgA (sIgA), are provided by the mother’s breast milk while the infant’s own production of intestinal sIgA by the gut-associated lymphoid tissue (GALT) develops [44,45]. In formula-fed infants, the immune protection provided by the mother is absent and the development of infant formula able to promote microbial colonization by *Bifidobacteria* and stimulate the development of immune competence is required. Three studies [14,18,27] assessed the effect of the probiotic on the development of mucosal immune competence using sIgA levels in the saliva or feces as a surrogate endpoint (Table 3). Among these studies, two were conducted in healthy infants (0–48 months old) [27] or healthy formula-fed babies (3–6 months old) [14], and one in children with non-infectious diarrhea (3–38 months old) [18].

Overall, supplementation with the probiotic increased or maintained higher sIgA levels, suggesting an enhanced mucosal immunity. Chen et al. (2007), in a pilot-like study, reported a significant increase in salivary sIgA levels in children of all age groups compared to randomly selected age-matched controls [27]. The study conducted by Xiao et al. (2019) enrolling 132 formula-fed infants showed that formula-fed infants receiving the probiotic maintained significantly higher fecal sIgA levels at the end of the 4-week supplementation period compared to those receiving placebo, suggesting a positive effect of probiotics on intestinal sIgA production [14]. Similarly, Liu et al. (2015) reported a significant increase in salivary sIgA after 3 days of supplementation in children with non-infectious diarrhea [18]. They also assessed the levels of inflammatory markers; the pro-inflammatory cytokines IL-6 and IL-17 were significantly reduced in the probiotic group at the end of treatment compared to controls [18].

The modulation of inflammatory marker was also assessed in a post hoc analysis of a placebo-controlled safety study cohort of healthy 3–12-month-old children receiving the individual strains for 8 weeks [13,28]. De Andres et al. (2018) reported that the IL10/IL-12 anti-inflammatory ratio increased significantly with *B. infantis* Rosell^®^-33 but decreased with placebo [28]. Interestingly, the authors also reported that the microbiota composition in children receiving the probiotic strains was similar to the microbiota profile typical of a 4-month-old, un-weaned infant, while the profile in the placebo group corresponded to that seen during the weaning process with a decrease in different *Bifidobacterium* species, such as *B. bifidum* and *B. breve*, and an increase in genera seen in the more diversified microbiota of adults (*Bacteroides, Blautia, Clostridium, Coprococcus* and *Faecalibacterium*) [28].

Regulation of immune function was also assessed using circulating levels of IgA in the serum. Although less is known about the role of serum IgA in regulating the systemic inflammatory response, low levels of serum IgA compared to other serum immunoglobulins have been observed in children with selective IgA deficiency, who are known to be more susceptible to sinopulmonary infections, GI illnesses, and allergic diseases [46,47]. Two studies conducted in Serbia [29,32] assessed the effect of this formulation on serum immunoglobulin levels in infection-prone children with low serum IgA levels. In both studies, most of the enrolled children were classified as atopic based on a positive sensitization response to 25–30 allergens in a skin prick test and/or elevated IgE levels. Pantovic et al. (2013) reported a normalization of serum IgA levels in 35% of the children after 3 months and 81% after 6 months [29]. Stoijkovic et al. (2016) reported a statistically significant decrease in IgE after 9 months of supplementation, and a rise in serum IgA, IgG, and IgM levels after 3 months of supplementation, which was maintained over the following 6 months [32]. While Pantovic et al. (2013) did not correlate the effects of the probiotic on serum IgA levels with the incidence of infections, two studies, including Stojkovic et al. (2016), have shown the efficacy of this probiotic against respiratory infections in frequently sick children.

### 3.4. Studies on the Prevention of Common Infections

Cazzola et al. 2010 [31] and Stojkovic et al. 2016 [32] investigated the efficacy of the probiotic (administered from 3 to 9 months) at preventing common respiratory infections or complications in children, such as upper respiratory tract infections (URTI), ear/nose/throat infections (ENTI), and GI illnesses. In France, Cazzola et al. (2010) enrolled 135 children diagnosed with >3 infections in the past winter to receive either probiotic (*n* = 62) or placebo (*n* = 73) for 3 months. There was a significant reduction of 24.7% (*p* ≤ 0.045) in the relative risk (RR) of occurrence of common infectious diseases in the probiotic group, with 51.6% (32/62) of children reporting at least one infection versus 68.5% (50/73) in the placebo group. Furthermore, 25.8% in the probiotic group (*p* ≤ 0.043) reported missing at least one school day for sickness versus 42.5% in placebo, representing a 40% decrease in school absenteeism [31]. Stojkovic et al. (2016) supplemented probiotics for 9 months in 78 hospitalized children aged 1.5 months to 5 years. All children were categorized into three groups according to the predominance of symptoms over the past year; Group I—predominance of respiratory infection and wheezing; Group II—predominance of respiratory infection without wheezing; Group III—predominance of wheezing without accompanying respiratory infection. The elevation in serum IgA levels in children from Groups I and II described above was correlated with a decline of clinical symptoms’ occurrence. In children from Group III a significant rise in serum IgA levels was observed only within 6–9 months and was correlated with a statistically significant decrease in the frequency of wheezing episodes at the end of the study [32]. This open-label before–after study shows that supplementation for 3–9 months results in a positive impact on the inflammatory status in children that is consistent with the improvement in clinical symptoms.

### 3.5. Studies in Other Indications

The studies classified in the category “other indications” are described below and summarized in Table 4.

#### 3.5.1. Oral Candidiasis (Thrush)

A Chinese study tested the probiotic for oral candidiasis infections (oral thrush) in 70 children (1–26 months old) [33]. The children were randomized into two groups of 35 receiving 2% sodium bicarbonate with nystatin (standard of care) with or without probiotic. Both the effective rate (94.3% vs. 77.1%, *p* < 0.05) and the recurrence rate (2.9% vs. 17.1%, *p* < 0.05) significantly favored the probiotic arm. The authors concluded that the probiotic was effective as adjuvant to nystatin and sodium bicarbonate in the treatment and prevention of oral thrush in children.

#### 3.5.2. Eczema

Li (2017) conducted a randomized controlled study to assess the effect of the probiotic (2 weeks) as an adjuvant to a standard topical treatment in 76 children with eczema (38/group) [34]. Baseline characteristics were similar between groups (*p* > 0.05). The Eczema Area and Severity Index (EASI) scores were significantly improved in the probiotic group compared to controls at 1 and 2 weeks after treatment (*p* < 0.05). The total effective rate in the intervention group was 92.1% (35/38) compared to 65.7% (25/38) in controls (*p* < 0.01).

#### 3.5.3. Iron Deficiency Anemia

Three studies of similar design investigated the effect of the probiotic as adjuvant to iron dextran supplementation for 8 weeks in a total of 182 children (6–60 months old) hospitalized for nutritional iron deficiency anemia (IDA) [35,36,37]. In parallel, children randomized to the control arms (*n* = 182) received iron dextran supplementation (standard of care) without probiotic. In all studies, the average age of enrolled children was approximately 3 years old, and the baseline characteristics were found similar between study arms in terms of age, gender distribution and severity of anemia (*p* > 0.05). Similar parameters to determine the efficacy of supplementation (i.e., blood anemia parameters) were used, including blood hemoglobin levels, mean corpuscular volume, mean corpuscular hemoglobin concentrations, reticulocyte counts, and serum iron levels. Treatment efficacy was measured as rates using a categorical scale, with a “markedly effective” result characterized by all symptoms and blood indicators returning to normal levels, an “effective” result characterized by an improvement in symptoms accompanied by a rise in hemoglobin levels by more than 20 g/L above baseline levels, and an “ineffective” result, by the absence of improvement in symptoms or by hemoglobin levels not increasing by more than 20 g/L above baseline levels [35,36,37].

**Figure 4:**
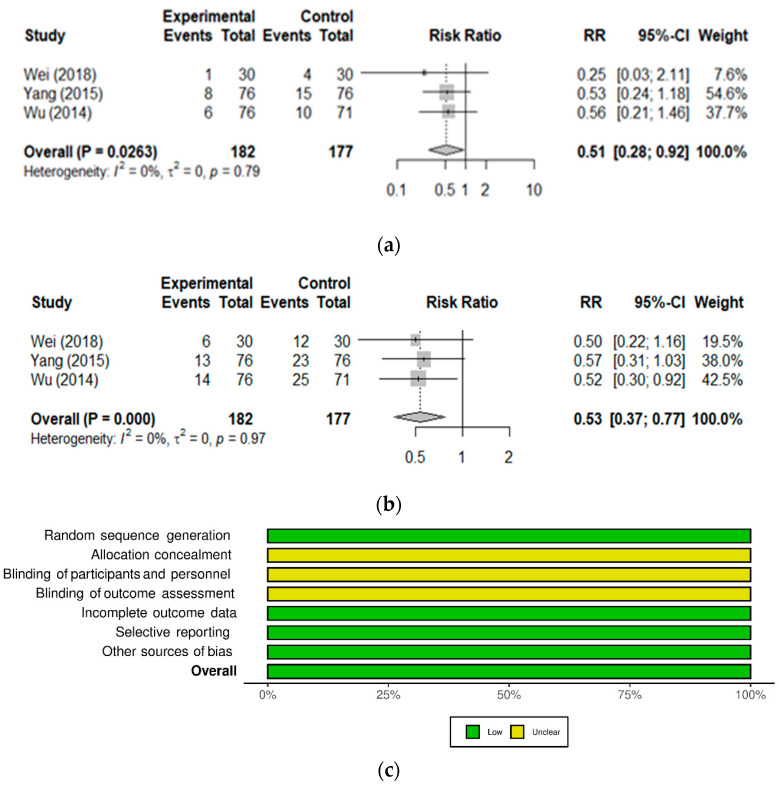
The probiotic reduced the relative risk of unresolved anemia and side effects in children with nutritional iron deficiency anemia receiving iron supplementation. Forest plot of studies on IDA treatment efficacy and occurrence of side effects. The 3 studies included in these meta-analyses are detailed in Table 4. (References: Wei, 2018 [35], Yang, 2015 [37], Wu, 2014 [36]). (**a**) Events refers to the number of cases categorized as inefficient (negative outcome of treatment); Total refers to the number of participants in the Experimental (Probiotic) and Control groups. (**b**) Events refers to the number of cases experiencing iron supplementation-related side effects; Total refers to the number of participants in the Experimental (Probiotic) and Control groups. (**c**) Mosaic plot showing the summary of the risk of bias analysis of studies included in the meta-analysis.

To compare these studies, ineffective cases were used to calculate the RR of treatment failure in the absence of probiotic (Figure 4a). When combining all three studies to increase the number of participants, the probiotic significantly reduced the risk of unresolved anemia by 49% (RR 0.51 [0.28–0.92]; *p* = 0.0263). Considering the high efficiency of iron dextran supplementation on its own, improvement of treatment efficacy with the probiotic (i.e., promoting iron absorption) is considered clinically meaningful. Furthermore, the common adverse effects of iron dextran supplementation, namely GI symptoms (diarrhea or constipation), nausea, and metal taste, were monitored in all 3 studies and found consistently reduced with the probiotic (Figure 4b), with a 47% reduction in the risk of side effects (RR 0.53 [0.37–0.77]; *p* = 0.0009) [35,36,37]. All three studies were found at low risk of bias (Figure 4c), except for the domains related to blinding which are typically not reported possibly due to regional reporting practices, as observed previously [41].

#### 3.5.4. GI Function and Necrotizing Enterocolitis in Newborns

In a randomized placebo-controlled trial, Huang et al. (2015) evaluated the safety and efficacy of the probiotic during 2 weeks on the GI function of 60 very low birth weight (VLBW) newborns (*n* = 30/arm; 1000–1500 g; average 34 weeks gestational age) [38]. All participants received intravenous nutrition starting on the second day after birth. Although not found statistically different, the incidence of necrotizing enterocolitis (NEC) was lower in the probiotic versus controls (6.7% vs. 16.7%; *p* > 0.05) with a relatively shorter hospital stay duration compared to controls (40.1 ± 15.6 vs. 47.3 ± 16.7 days; *p* > 0.05). Adverse events were similar between groups, with one death (3.3%) in the probiotic group and two (6.7%) in the controls. There was a slightly lower incidence of parenteral nutrition-associated cholestasis (PNAC) in the probiotic group with only 1 case (3.3%) vs. 3 cases (10%) in controls (*p* > 0.05). Feeding intolerance, which was assessed by the occurrence of GI symptoms (i.e., vomiting, diarrhea, or constipation), was significantly lower in the probiotic vs. controls (36.7% vs. 70%; *p* = 0.0104). Results suggest a positive effect of this formulation on the GI function in VLBW infants, which supports the conduct of further clinical trials assessing the effect of this probiotic formulation on the incidence and severity of NEC and PNAC in larger cohorts [38].

#### 3.5.5. Jaundice in Newborns

In a double-blind, placebo-controlled trial, Gao et al. (2015) assessed the safety and efficacy of a 7 day prophylaxis with the probiotic for the prevention of jaundice in a population of 1000 healthy full-term neonates (*n* = 500/group; 2500–4000 g; 37–42 weeks gestational age) [39]. The time of onset of jaundice was similar between groups (*p* > 0.05), but incidence was significantly lower (*p* < 0.001) in probiotics (9%) versus controls (17%). The average levels of daily percutaneous bilirubin from the second to fifth postnatal day were similar in both groups (*p* > 0. 05) but the average levels of daily percutaneous bilirubin on the sixth and seventh day after birth were significantly lower in probiotics versus controls (*p* < 0.05). Adverse events were similar between groups (*p* > 0.05). This probiotic formulation was well tolerated, clinically safe, and reduced the daily percutaneous bilirubin levels and incidence of hyperbilirubinemia in newborns [39].

Qiu et al. (2020) conducted a study on the efficacy of the probiotic as an adjuvant to blue light phototherapy (450–475 nm; light intensity 10–12 µW/cm^2^/nm) in 64 full-term otherwise healthy newborns (gestational age 37–41 weeks) diagnosed with jaundice (*n* = 32/arm) [30,48]. Briefly, only children younger than 3 days old, in whom appearance of jaundice occurred within 24 h from birth, with a total bilirubin level above 102 µmol/L, a daily rise in bilirubin above 85 µmol/L, and conjugated bilirubin levels above 25 µmol/L were included. Exclusion criteria were congenital biliary malformations, acute bilirubin encephalopathy with central nervous system (CNS) manifestations, a score lower than 7 on the Agpar test (one minute), cytomegalovirus infections, or severe infections (e.g., septic shock, CNS infections). Although jaundice improved in both groups, there was a significantly higher reduction in the total and conjugated bilirubin levels at 3 and 5 days of treatment in the probiotic group (*p* < 0.05). There was also a significant reduction in IL-6 levels in the probiotic group compared to controls (*p* < 0.05) and a significant reduction in IL-6 and IL-8 levels at day 3 and 5, and a significant increase in IL-10 at day 5 vs. baseline in the probiotic group (all *p* < 0.05), but not in the controls. Oxidative stress markers were unchanged in both groups, except for serum catalase levels at day 5, which were significantly higher in the probiotic group compared to baseline (*p* < 0.05) [30]. Overall, the probiotic formulation was found safe and effective as an adjunct to blue light phototherapy for jaundice in otherwise healthy full-term newborns, possibly by improving the inflammatory status or, more speculatively, oxidative stress levels.

## 4. Discussion and Concluding Remarks

The clinical studies reviewed herein demonstrate the efficacy of this formulation as an adjuvant to standard treatment for diarrhea of various etiologies [15,16,17,18,19,20,21,22,23,24,25,26] and support its role at sustaining natural defenses against infections and boosting immunity [13,14,15,17,18,19,21,22,23,24,25,26,28,29,30,31,32]. Promising results support additional studies on this probiotic formulation in children with oral candidiasis [33], iron deficiency anemia [35,36,37], eczema [34], as well as for preventing jaundice or improving GI function in newborns [30,38,39].

The safety of this formulation has been confirmed in several studies and through the pharmacovigilance program in place [7]. While adverse events monitoring is expected to have been conducted in all trials, some studies have not adequately reported on this aspect. However, in the 13 studies where adverse events monitoring was reported [13,14,15,20,31,32,33,34,35,36,37,38,39] few adverse events were observed, and none could be attributed to the intervention over placebo. While the systematic reporting of adverse events in clinical trials generally needs to be improved, there is no evidence of increased risk resulting from probiotic use in otherwise healthy children [49]. However, caution is generally recommended for all probiotics in individuals more susceptible to bacterial infections, such as children younger than 3 years old with short bowel syndrome or in severely immunocompromised individuals and immunosuppressed patients [50,51]. The innocuity of the strains in this formulation is recognized by several authoritative bodies worldwide, where the strains are included in lists of safe strains for consumption in foods. Probiokid^®^ and the individual strains have been recognized for their safety by the US FDA in the form of a no question letter for notified GRAS status for use in non-exempt infant formula, and in Canada from the NNHPD for use in Natural Health Products in infants 3 months old and over.

This review has strengths and limitations. A strength of this review is the cohesion between studies allowing to conduct outcome-specific meta-analyses without heterogeneity. Indeed, several meta-analyses attempt to compare studies using several different probiotics and measuring clinical outcomes with a variety of methods. Here, the studies are homogeneous in terms of probiotic intervention, study designs and outcome assessment methods, which strengthens the conclusions reached by individual studies, most notably with regard to the benefits on diarrhea treatment. Except for 2 studies that did not provide details on the diagnosis criteria used to define acute diarrhea, the 10 remaining studies used local guidelines in place for the diagnosis of diarrheal diseases. Another limitation of this review is the fact that many studies were assigned an unclear of high risk of bias. In many studies, while randomization was used, reporting was deficient in terms of the description of the randomization method, allocation concealment or blinding procedures implemented. This could potentially be explained by the existence of country-specific reporting guidelines. Nevertheless, the flow of participants throughout the studies and clinical outcomes were well described, which suggests a generally low risk of attrition and reporting biases in outcomes.

For the meta-analysis of IDA studies, the risk of bias was found low in all domains, heterogeneity was absent, and the results were identical when fixed- or random-effects models were applied. However, it is known that standard random-effects meta-analyses with few studies (*n* = 2 or 3) are less accurate, especially in the presence of heterogeneity or when studies are highly variable in size [52]. Hence, while the current positive effects in children with IDA receiving iron supplementation are promising, the conclusion about the efficacy of the formulation in this specific indication could change with an increased number of studies.

This probiotic formulation was mostly studied in children. However, a similar effect of the strain *B. bifidum* Rosell^®^-71 was shown in healthy young adults undergoing university exams; this strain significantly increased the proportion of healthy days (i.e., without cold and flu symptoms) and reduced diarrhea symptoms associated with stress [53,54]. Mechanistic insights on the modes of action of this probiotic formulation were obtained from in vitro and in vivo studies. Briefly, the formulation and individual strains were shown to preserve the integrity of the intestinal barrier, and to exert immunomodulatory functions in vitro and in vivo, which may also contribute to the beneficial effects observed on diarrhea symptoms and overall GI and immune health. In vitro, this probiotic was shown to counteract Toll-like receptor 3 (TLR3)-induced inflammation triggered by the double-stranded RNA ligand Poly(I:C), which mimics a Th1 and antiviral innate immune response in human intestinal epithelial cells [55,56]. In vivo, the formulation induced mucin and tight junction protein expression, and suppressed the inflammation and apoptosis induced by the carcinogenic compound dimethylhydrazine dihydrochloride [57]. Furthermore, this probiotic increased anti-inflammatory cytokines (i.e., IL-4) and reduced pro-inflammatory cytokines (e.g., IL-1a, IL-1b, IL-6, IFN-γ and TNF-α) in rats infected with enterotoxic *E. coli* (model of a Th1 immune response), and reduces pro-inflammatory cytokines (e.g., IL-1a) in rats infected with the pathogen *N. brasiliensis* (model of a Th2 immune response) [58].

In addition to regulating host defenses, probiotics’ effects on the immune systems also influence the host in a systemic manner. Recently, an in vivo study on this probiotic formulation revealed that the immunomodulatory effects of this formulation could also underly a potential role in indications related to the microbiota–gut–brain axis (MGBA). Indeed, this probiotic formulation prevented the increase in pro-inflammatory cytokines in the dam’s serum and fetal brain in the maternal immune activation (MIA) mouse model of autism [59]. In addition, it prevented the neuronal loss and the reduction in GABA levels seen in the MIA adult offspring’s prefrontal cortex. These changes were correlated with behavioral improvements compatible with an amelioration in autism-like symptoms in these mice (social deficits and stereotypical behavior), but also reduced anxiety- and depression-related behaviors in the adult MIA offspring. This study, by linking the immunomodulatory and behavioral effects of Probiokid^®^ with the microbiota–gut–brain axis in a pre-clinical setting, provides a rationale to explore the potential role of this formulation in other MGBA models. Of note, the limited number of clinical trials conducted so far in children with ASD have resulted in conflicting results about the potential of probiotics to improve GI or behavioral symptoms in this population [60]. In addition, the beneficial effects on GI function with a trend towards a reduction in NEC incidence in a small cohort of premature infants [38], along with the reduction in the severity of intestinal tissue damage in a rodent model of NEC [61] warrant further studies on this probiotic in premature infants at risk of NEC.

Overall, the current knowledge base available on this probiotic indicates its efficacy to produce clinical benefits in various indications frequently seen in children, including diarrhea of various etiology and respiratory or other infections. The promising positive effects seen in a variety of conditions commonly affecting newborns and children warrants more studies on this probiotic to better understand its mechanisms of action in immune modulation and gastrointestinal protection, but also at the systemic level based on pre-clinical data. However, there is currently a gap in knowledge about how microbiota-derived molecules may systemically affect the host, especially considering the complexity of these interactions and variable translatability of mechanisms from animal models to humans. The recent advents in “meta-omics” technologies and use of various experimental models [62,63,64,65] could help bridge this gap.

## Figures and Tables

**Table 1:** Overview of the clinical studies in pediatric populations.

Indication	Number of Studies	N (Total)	N (Probiotic Arm)	Main Outcome Measures	References
**Safety**					
Healthy children/newborns	2	340	≈50/strain [13] ^1^ 66 [14]	Growth parameters, adverse events and serious adverse events, sleep and crying patterns, D-lactic acid	[13,14]
**Gastrointestinal function**					
Non-infectious diarrhea	7	1001	521	Time to symptom relief, effective rate	[15,16,17,18,19,20,21]
Rotavirus (RV)-induced diarrhea	4	394	207	Time to symptom relief, effective rate	[22,23,24,25]
Persistent diarrhea, undefined etiology	1	52	32	Time to symptom relief, effective rate	[26]
**Immune system function and natural defenses**					
Secretory IgA, cytokines, chemokines	6	405 ^2^	224 ^2^	Salivary, fecal or serum levels of secretory IgA, fecal or serum levels of cytokines/chemokines	[14,18,27,28,29,30]
Common infections ^3^	2	213	140	Incidence of infections and related symptoms, adverse events	[31,32]
**Other indications**					
Oral candidiasis (thrush)	1	70	35	Effective rate, recurrence	[33]
Eczema	1	76	38	Eczema Area and Severity Index (EASI) score, effective rate	[34]
Iron deficiency anemia	3	364	182	Anemia blood markers, effective rate, side effects	[35,36,37]
Necrotizing enterocolitis	1	60	30	Incidence, severity, mortality, food tolerance	[38]
Jaundice	2	1064	532	Incidence, severity (bilirubin levels)	[30,39]

^1^ Study assessing individual strains. ^2^ Not including children from De Andres et al. (2018) [28], a post hoc analysis of Manzano et al. (2007) [13] assessing single strains vs. placebo (≈50/strain). ^3^ Including upper respiratory tract infections, ear-nose-throat infections, and gastrointestinal symptoms.

**Table 2:** Details of studies on diarrhea of various etiologies.

Reference [Language]	Study Dates	Population	Study Design Arms, *n*	Probiotic Regimen	Results (vs. Control)	Adverse Events
Cui, 2003 [22] [Chinese]	September 2002–November 2002	Children 6–24 months old RV-ag^+^ diarrhea Onset < 72 h	Randomized, controlled Ribavirin + Pro, *n* = 62 Ribavirin + Lacidophilin, *n* = 60	<12 months: 1 sachet QD 12–24 months: 1 sachet BID Orally in warm water or milk Until resolution or up to 72 h	Shorter duration of diarrhea (39.3 ± 17.1 vs. 63.8 ± 22.9 h) Higher total effective rate (93.5% vs. 61.7%; *p* < 0.01)	n.r.
Li, 2008 [24] [Chinese]	June 2005–December 2007	Children 0–60 months old RV-ag^+^ diarrhea	Randomized, controlled Ribavirin + Pro, *n* = 39 Ribavirin alone, *n* = 39	1 sachet BID Orally. 7 days	Higher total effective rate (94.9% vs. 74.3%; *p* < 0.05)	n.r.
Jiang, 2008 [26] [Chinese]	December 2006–June 2008	Children 3–24 months old Persistent diarrhea	Randomized, active control Pro, *n* = 32 GoldenBifido, *n* = 20	<6 months: 0.5 sachet BID 6–12 months: 1 sachet BID 12–24 months: 1–2 sachet BID Until resolution	Shorter duration of diarrhea (7.14 ± 0.78 vs. 12.6 ± 1.75 d; *p* < 0.001) Higher total effective rate (91% vs. 65%; *p* < 0.01)	n.r.
Yang, 2010 [25] [Chinese]	January 2008–October 2009	Children 6–30 months old RV-ag+ diarrhea	Randomized, controlled CST + Pro (in formula), *n* = 58 CST (breastfed or formula), *n* = 40	1 sachet QD Orally In lactose-free formula Duration not stated	Shorter duration of diarrhea (2.8 ± 1.1 vs. 4.9 ± 2.6 d; *p*< 0.01) Shorter hospital stay (5.5 ± 1.7 vs. 8.5 ± 2.3 d; *p* < 0.01). Higher total effective rate (94.8% vs. 77.5%; *p* ≤ 0.05)	n.r.
Wang, 2012 [20] [Chinese]	May 2010–December 2010	Children 3–36 months old Non-infectious diarrhea Onset < 72 h	Randomized, controlled Smecta^®^ + Pro, *n* = 104 Smecta^®^, *n* = 90	<12 months: 0.33 sachet TID 12–24 months: 0.5 sachet BID 24–36 months: 1 sachet BID Orally. 3 days	Higher markedly effective rate in three age groups (*p* > 0.05): < 12 months: 78.8% vs. 74.2% 12–24 months: 79.1% vs. 74/3% 24–36 months: 82.1% vs. 75%	None observed
Luo, 2013 [19] [Chinese]	April 2010–February 2011	Children 4–36 months old Non-infectious diarrhea Onset < 72 h	Randomized, controlled Smecta^®^ + Pro, *n* = 75 Smecta^®^, *n* = 68	<12 months: 0.33 sachet TID 12–24 months: 0.5 sachet BID 24–36 months: 1 sachet BID Orally. 3 days	Higher effective rate (94.8% vs. 73%; *p* < 0.05)	n.r.
Gao, 2013 [15] [Chinese]	January 2011–January 2012	Children 0–36 months old Non-infectious diarrhea	Randomized, controlled Smecta^®^ + Pro, *n* = 43 Smecta^®^, *n* = 43	<12 months: 0.33 sachet TID 12–36 months: 0.5 sachet TID Orally. 3 days	Higher total effective rate (90.7% vs. 62.8%; *p* < 0.05)	None observed
Wu, 2013 [21] [Chinese]	April 2011–December 2011	Children 2–36 months old Non-infectious diarrhea Onset < 72 h	Randomized, controlled Smecta^®^ + Pro, *n* = 84 Smecta^®^, *n* = 64	<12 months: 0.33 sachet TID 12–24 months: 0.5 sachet BID 24–36 months: 1 sachet BID Orally. 3 days	Higher total effective rate (90.5% vs. 75%; *p* < 0.05)	n.r.
Jin, 2014 [23] [Chinese]	October 2011–June 2013	Children 5–52 months old (mean 17.3 mo) RV-ag^+^ diarrhea. Onset < 72 h	Randomized, active control Smecta^®^ + Ribavirin + Pro, *n* = 48 Smecta^®^ + Ribavirin, *n* = 48	0.5–1 sachet BID Orally. In warm water. Duration not stated. Fluids provided as needed	Shorter time to symptom relief (diarrhea, 31.6 ± 5.2 h vs. 34.6 ± 4.1 h; *p*< 0.05) Higher total effective rate (91.7% vs. 75.0%; *p* < 0.05)	n.r.
Liu, 2015 [18] [Chinese]	May 2011–May 2014	Children 3–38 months old Non-infectious diarrhea	Randomized, controlled Smecta^®^ + Pro, *n* = 75 Smecta^®^, *n* = 75	<12 months: 0.33 sachet TID 12–24 months: 0.5 sachet BID 24–36 months: 1 sachet BID Orally. 3 days	Higher markedly effective rate (72% vs. 45.33%; *p* < 0.05) Higher total effective rate (96% vs. 84%; *p* < 0.05) Reduced diarrhea frequency (*p* < 0.05), time to diarrhea relief (*p* < 0.05) and symptom disappearance (*p* < 0.05) in the probiotic group	n.r.
Liang, 2018 [17] [Chinese]	February 2015–May 2017	Children 3–39 months old Non-infectious diarrhea	Randomized, active control Smecta^®^ + Probiotic, *n* = 43 Smecta^®^ alone, *n* = 43	<12 months: 0.33 sachet TID 12–24 months: 0.5 sachet BID 24–36 months: 1 sachet BID Orally. 3 days	Higher total effective rate (95.35% vs. 79.07%; *p* < 0.05)	n.r.
Li, 2020 [16] [Chinese]	January 2019–February 2020	Children 6–37 months old Non-infectious diarrhea	Randomized, controlled Smecta^®^ + Pro, *n* = 49 Smecta^®^, *n* = 49	<12 months: 0.33 sachet TID 12–24 months: 0.33 or 0.66 sachet TID 24–36 months: 1 sachet TID Orally. 3 days	Higher total effective rate (95.91% vs. 83.67%; *p* < 0.05)	n.r.

BID, twice a day; CST, comprehensive standard therapy (as needed; Smecta^®^, fluids, antibiotics, etc.); n.r., not reported; Pro, Probiotic (Biostime^®^); RV-ag^+^, positive for fecal rotavirus antigen; TID, three times per day; QD, once daily.

**Table 3:** Details of studies on immunity and natural defenses.

Reference [Language]	Study Dates	Population	Study Design Arms, *n*	Probiotic Regimen	Results (vs. Control)	Adverse Events
Chen, 2007 [27] [Chinese]	Not stated	Children 0–48 months old Healthy with low salivary sIgA	Randomized, controlled Probiotic, *n* = 20 No Intervention, *n* = 8	1 sachet BID Orally. For 14 days	Increase in salivary sIgA compared to baseline in probiotic but not in controls. No statistical analyses reported.	n.r.
Cazzola, 2010 [31] [English]	December 2006–March 2007	Children 3–7 years old ≥ 3 infections (ENTI, URTI, GI illness) in past winter.	Randomized, double-blind, placebo-controlled Probiotic, *n* = 62 Placebo, *n* = 73	1 sachet QD Orally. For 3 months	Lower incidence of ENTI, URTI, or GI health events (51.6% vs. 68.5%; *p* = 0.044), representing a 25% reduction in RR. Less participants experienced school day losses for sickness (25.8% vs. 42.5%; *p* = 0.0443).	2 SAEs; 1 abdominal pain in placebo and 1 otitis media in Probiotic. 24 AEs in 20 children (9 in placebo, 11 in Probiotic), most were expected respiratory or GI events.
Pantovic, 2013 [29] [English]	Not stated	Children 6–42 months old low IgA levels hospitalized for URTI or ENTI	Open-label, uncontrolled before–after study Probiotic, *n* = 31	1 sachet QD Orally. For 6 months	Increase in serum IgA levels in 35% of the children after 3 months and 81% after 6 months (*p* < 0.05), normalized to normal range. Clinical improvement in URTI after 3 months, and no infections diagnosed between 3 and 6 months.	n.r.
Liu, 2015 [18] [Chinese]	May 2011–May 2014	Children 3–38 months old Non-infectious diarrhea	Randomized, controlled Smecta^®^ + Probiotic, *n* = 75 Smecta^®^, *n* = 75	<12 months: 0.33 sachet TID 13–24 months: 0.5 sachet BID 24–36 months: 1 sachet BID Orally. For 3 days	Lower serum levels of pro-inflammatory cytokines IL-6 and IL-17 (*p* < 0.05). Higher levels of salivary sIgA (*p* < 0.05).	n.r.
Stojkovic, 2016 [32] [English]	Not stated.	Children <5 years Hospitalized during the past year for respiratory diseases	Open label, before–after Probiotic, *n* = 78 Divided into 3 groups based on medical history: G1: URTI + wheezing, *n* = 50 G2: URTI w/o wheezing, *n* = 17 G3: Wheezing w/o URTI, *n* = 11	1 sachet QD Orally. For 9 months	Decrease in URTI and wheezing after 3 months (*p* < 0.01), reaching 0% after 6 months. No recurrence of URTI or wheezing (0%) in all groups) at 9 months. Increase in serum IgA from 3 months onwards (*p* < 0.01). Increase in serum IgG from 3 months onwards (*p* < 0.01). Decrease in serum IgE at 9 months (*p* < 0.01).	None observed.
Manzano, 2017 [13]; De Andres, 2018 [28] [English]	August 2014–December 2016	Children 3–12 months old Healthy	Randomized, double-blind, placebo-controlled *L. helveticus* Rosell^®^-52, *n* = 52 *B. infantis* Rosell^®^-33, *n* = 53 *B. bifidum* Rosell^®^-71, *n* = 51 Placebo, *n* = 52	3 × 10^9^ CFU of each single strain QD Orally. For 8 weeks	Increased IL10/IL12 ratio (anti-inflammatory) in *B. infantis* (*p* < 0.01). Increased TNFα/IL10 ratio (pro-inflammatory) in *L. helveticus* and placebo (*p* < 0.01). Placebo group showed a microbiota composition related to the weaning process, while the probiotics groups were similar to 4-month-old un-weaned infants.	No difference between groups for the number and severity of adverse events (mild) nor in behavioral and anthropometric parameters. No SAEs observed.
Xiao, 2019 [14] [English]	December 2014–November 2015	Children 3.5–6 months old Healthy	Randomized, placebo-controlled Probiotic, *n* = 66 Placebo, *n* = 66	1 sachet QD Orally, in formula. For 4 weeks	Maintained higher fecal sIgA levels at the end of the four-week treatment period (*p* < 0.05).	All AEs reported were minor and more frequent in the placebo group. Probiotic: 37 AEs; 21 respiratory, 12 GI, 4 dermatological. Placebo: 69 AEs; 38 respiratory, 15 GI, 16 dermatological. No effect on growth rate.
Qiu, 2020 [30] [Chinese]	September 2017–May 2018	Full-term neonates with hyperbilirubinemia	Randomized, controlled Blue light photo- therapy + Probiotic, *n* = 32 Blue light photo- therapy, *n* = 32	1 sachet BID, for 5 days	Reduction in IL-6 vs. controls (*p* < 0.05). Reduction in IL-6 and IL-8 at day 3 and 5 vs. baseline (*p* < 0.05). Increase in IL-10 at day 5 vs. baseline (*p* < 0.05). Increase in serum catalase levels at day 5 vs. baseline (*p* < 0.05).	n.r.

BID, twice a day; CFU, colony-forming unit; ENTI, ear-nose-throat infection; GI, gastrointestinal; n.r., not reported; QD, once daily; sIgA, secretory immunoglobulin A; TID, three times per day; URTI, upper respiratory tract infection.

**Table 4:** Details of studies in other indications.

Reference [Language]	Study Dates	Population	Study Design Arms, *n*	Probiotic Regimen	Results (vs. Control)	Adverse Events
Xi, 2013 [33] [Chinese] (Thrush)	January 2011–December 2012	Children 1–26 months old oral candidiasis infection	Randomized, controlled 2% NaHCO₃ + nystatin + Pro, *n* = 35 2% NaHCO₃ + nystatin, *n* = 35	1 sachet BID Orally. For 14 days	Higher total effective rate (94.3% vs. 77.1%, *p* ≤ 0.05). Lower recurrence rate (2.9% vs. 17.1%, *p* ≤ 0.05).	None observed.
Li, 2017 [34] [Chinese] (Eczema)	August 2014–January 2016	Children 1–24 months old infantile eczema	Randomized, controlled Topical treatment + Probiotic, *n* = 38 Topical treatment, *n* = 38	1 sachet BID, for 2 weeks	Lower EASI scores at 2 weeks after treatment (*p* < 0.05). Higher total effective rate (92.1% vs. 65.7%, *p* < 0.01).	None observed.
Wu, 2014 [36] [Chinese] (IDA)	June 2010–April 2013	Children 6–60 months old Nutritional iron deficiency anemia	Randomized, controlled Iron dextran, *n* = 71 Iron dextran + Probiotic, *n* = 76	1 sachet QD, for 8 weeks	Higher total effective rate (76.3% vs. 60.6 %; χ^2^ = 4.236, *p* = 0.040).	Decreased cases of side effects of Iron dextran (13.2% vs. 25.4%; *p* = 0.06).
Yang, 2015 [37] [Chinese] (IDA)	February 2011–December 2013	Children 6–60 months old Nutritional iron deficiency anemia	Randomized, controlled Iron dextran, *n* = 76 Iron dextran + Probiotic, *n* = 76	1 sachet BID, for 8 weeks	Higher markedly effective rate (75% vs. 55.3%; χ^2^ = 6.453, *p* = 0.011).	Decreased cases of side effects of iron dextran (17.1% vs. 30.3%; *p* = 0.06).
Wei, 2018 [35] [Chinese] (IDA)	January 2015–December 2016	Children 7–72 months old Nutritional iron deficiency anemia	Randomized, Controlled Iron dextran, *n* = 30 Iron dextran + Probiotic, *n* = 30	1 sachet BID, for 8 weeks All received also Smecta^®^ and antibiotics	Total effective rate 10% higher in probiotic vs. control (96.7% vs. 86.7%; *p* > 0.1).	Decreased cases of side effects of iron dextran (20% vs. 40%; *p* = 0.09).
Huang and Ouyang, 2015 [38] [Chinese] (GI function; NEC)	August 2011–August 2013	Premature newborns (1000–1500 g; mean 34 weeks gestational age)	Randomized, placebo-controlled Placebo, *n* = 30 Probiotic, *n* = 30	0.5 sachet BID, for 2 weeks	Reduced feeding intolerance vs. control (36.7% vs. 70%; *p* = 0.0104). Nonsignificant reduction in NEC incidence in probiotic (2/30; 6.7%) vs. control (5/30; 16.7%). Nonsignificant reduction in duration of hospital stay in probiotic (40.1 ± 15.6 d) vs. control (47.3 ± 16.7 d).	One death (3.3%) in the probiotic group, 2 deaths in the control group (6.7%). Parenteral nutrition-associated cholestasis: 1 case (3.3%) in probiotic vs. 3 cases (10%) in control (*p* = 0.2755).
Gao, 2015 [39] [Chinese] (Jaundice)	December 2013–December 2013	Healthy full-term neonates gestational age 37–42 weeks (mean 40 weeks), birth weight 2500–4000 g	Randomized, placebo-controlled Placebo, *n* = 500 Probiotic, *n* = 500	1 sachet BID, for 7 days after birth	Similar time of onset of jaundice between groups (*p* > 0.05), with significantly lower incidence in probiotic (9%) vs. placebo (17%); *p* < 0.001. Similar daily percutaneous bilirubin levels until day 5 (*p* > 0.05), with a significant reduction in bilirubin levels in probiotic on treatment days 6 and 7 vs. placebo (*p* < 0.05).	Similar between groups (erythema, vomiting, diarrhea; all *p* > 0.05).
Qiu, 2020 [30] [Chinese] (Jaundice)	September 2017–May 2018	Healthy full-term neonates gestational age 37–41 weeks, aged ≤3 days, with neonatal hyperbilirubinemia developing within 24 h of birth	Randomized, controlled Blue light phototherapy, *n* = 32 Blue light phototherapy + Probiotic, *n* = 32	1 sachet BID, for 5 days	Significant decrease in total and unconjugated bilirubin levels in probiotic vs. controls at day 3 and day 5 (*p* < 0.05). Significant increase in catalase levels vs. baseline at day 5 (*p* < 0.05) in the probiotic group, but no change in controls.	n.r.

BID, twice a day; IDA, iron deficiency anemia; NEC, necrotizing enterocolitis, n.r., not reported; QD, once daily; TID, three times per day.

## Supplementary Materials

The following are available online at https://www.mdpi.com/article/10.3390/nu13061908/s1, Table S1: PubMed search strategy.
