# Supplementary material for: Efficacy of a Multi-Strain Probiotic Formulation in Pediatric Populations: A Comprehensive Review of Clinical Studies
Source: Nutrients. 2021 Jun 1;13(6):1908. doi: 10.3390/nu13061908 (PMC8226750; doi:10.3390/nu13061908)
Supplement: Supplementary file 1 [file nutrients-13-01908-s001.zip › nutrients-1233278-supplementary.pdf]

Supplemental Table 1

**Detailed search strategy for PubMed:**
(("lactobacillus helveticus"[MeSH Terms] OR ("lactobacillus"[All Fields] AND "helveticus"[All Fields]) OR "lactobacillus helveticus"[All Fields]) AND ("bifidobacterium bifidum"[MeSH Terms] OR ("bifidobacterium"[All Fields] AND "bifidum"[All Fields]) OR "bifidobacterium bifidum"[All Fields]) AND ("bifidobacterium longum subspecies infantis"[MeSH Terms] OR ("bifidobacterium"[All Fields] AND "longum"[All Fields] AND "subspecies"[All Fields] AND "infantis"[All Fields]) OR "bifidobacterium longum subspecies infantis"[All Fields] OR ("bifidobacterium"[All Fields] AND "infantis"[All Fields]) OR "bifidobacterium infantis"[All Fields])) OR ("R0033"[All Fields] AND "R0052"[All Fields] AND "R0071"[All Fields]) OR ("Rosell-33"[All Fields] AND "Rosell-52"[All Fields] AND "Rosell-71"[All Fields]) OR "Biostime"[All Fields]
**Translations**
**Lactobacillus helveticus:** "lactobacillus helveticus"[MeSH Terms] OR ("lactobacillus"[All Fields] AND "helveticus"[All Fields]) OR "lactobacillus helveticus"[All Fields]
**Bifidobacterium bifidum:** "bifidobacterium bifidum"[MeSH Terms] OR ("bifidobacterium"[All Fields] AND "bifidum"[All Fields]) OR "bifidobacterium bifidum"[All Fields]
**Bifidobacterium infantis:** "bifidobacterium longum subspecies infantis"[MeSH Terms] OR ("bifidobacterium"[All Fields] AND "longum"[All Fields] AND "subspecies"[All Fields] AND "infantis"[All Fields]) OR "bifidobacterium longum subspecies infantis"[All Fields] OR ("bifidobacterium"[All Fields] AND "infantis"[All Fields]) OR "bifidobacterium infantis"[All Fields]
